# Oral manifestations of human papillomavirus infections

**DOI:** 10.1111/eos.12538

**Published:** 2018-09-03

**Authors:** Stina Syrjänen

**Affiliations:** ^1^ Department of Oral Pathology and Oral Radiology Institute of Dentistry Faculty of Medicine University of Turku Turku Finland; ^2^ Department of Pathology Turku University Hospital Turku Finland

**Keywords:** human papillomaviruses, oral infection, prevention, saliva, transmission

## Abstract

Papillomaviruses are one of the oldest viruses known, dating back 330 million years. During this long evolution, human papillomaviruses (HPV) have developed into hijackers of human cellular and immune systems in which they replicate and remain silent. Systematic studies on oral HPV infections and their outcomes are still scarce. Oral HPV infections have been linked to sexual behaviour, but recent evidence supports their horizontal, mouth‐to‐mouth, transmission. Most HPV infections in infants are acquired vertically from the mother during the intrauterine period, during delivery, or later via saliva. The best‐known benign clinical manifestations of HPV infection are oral papilloma/condyloma and focal epithelial hyperplasia. Evidence is emerging which suggests that some oral HPV infections might persist**.** Persistent HPV infection is mandatory for HPV‐associated malignant transformation. However, progression of HPV‐induced lesions to malignancy requires additional cofactors. In the early 1980s, we provided the first evidence that a subset of oral cancers and other head and neck cancers might be causally linked to HPV infection. This review summarizes current knowledge on the virus itself, its transmission modes, as well as the full spectrum of oral HPV infections – from asymptomatic infections to benign, potentially malignant oral lesions, and squamous cell carcinoma.

Papillomaviruses are the oldest existing viruses, originating from the late Paleozoic Era some 330 million years ago. Papillomaviruses have strict species‐specificity with a broad genotypic diversity. The ancient papillomaviruses with mucosal tropism started developing some 90 million years ago 1. During their evolution, human papillomaviruses (HPVs) developed, acquiring the capacity to utilize human cellular proteins for replication and to remain silent by hijacking the cellular and immune systems at several levels 2. The manifestations of HPV infections can be multiple, varying from asymptomatic infections to benign warty or potentially malignant lesions, intraepithelial neoplasia, and invasive carcinomas 3, 4, 5. At the time of writing, over 205 different HPV genotypes had been identified and were categorized into five genera, of which *Alphapapillomavirus*,* Betapapillomavirus*, and *Gammapapillomavirus* are the largest 6, 7. Human papillomaviruses in the alpha genus are of main clinical importance as this genus contains most of the mucosal HPVs, which include both so‐called low‐risk and high‐risk HPVs (Fig. 1) 8. The low‐risk mucosal HPVs, such as HPV‐6 and HPV‐11, cause benign papilloma/condyloma, whereas the high‐risk mucosal HPVs, such as HPV‐16 and HPV‐18, cause squamous intraepithelial lesions that can progress to squamous cell carcinoma in the head and neck region and/or anogenital tract.

**Figure 1:**
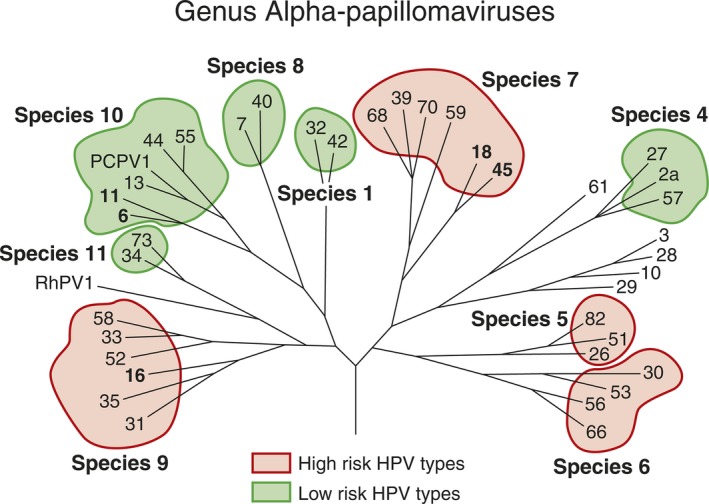
Human papillomavirus (HPV) genotypes among species of the genus *Alphapapillomavirus*. The species containing high‐risk and low‐risk HPV genotypes are marked with pink and green colors, respectively. Reprinted from Rautava J and Syrjänen S. Biology of Human Papillomavirus Infections in Head and Neck Carcinogenesis. Head Neck Pathol. 2012; 6(Suppl 1): 3–1. With permission from Springer Nature via Copyright Clearance Center's RightsLink service.

## HPV genome

**Figure 2:**
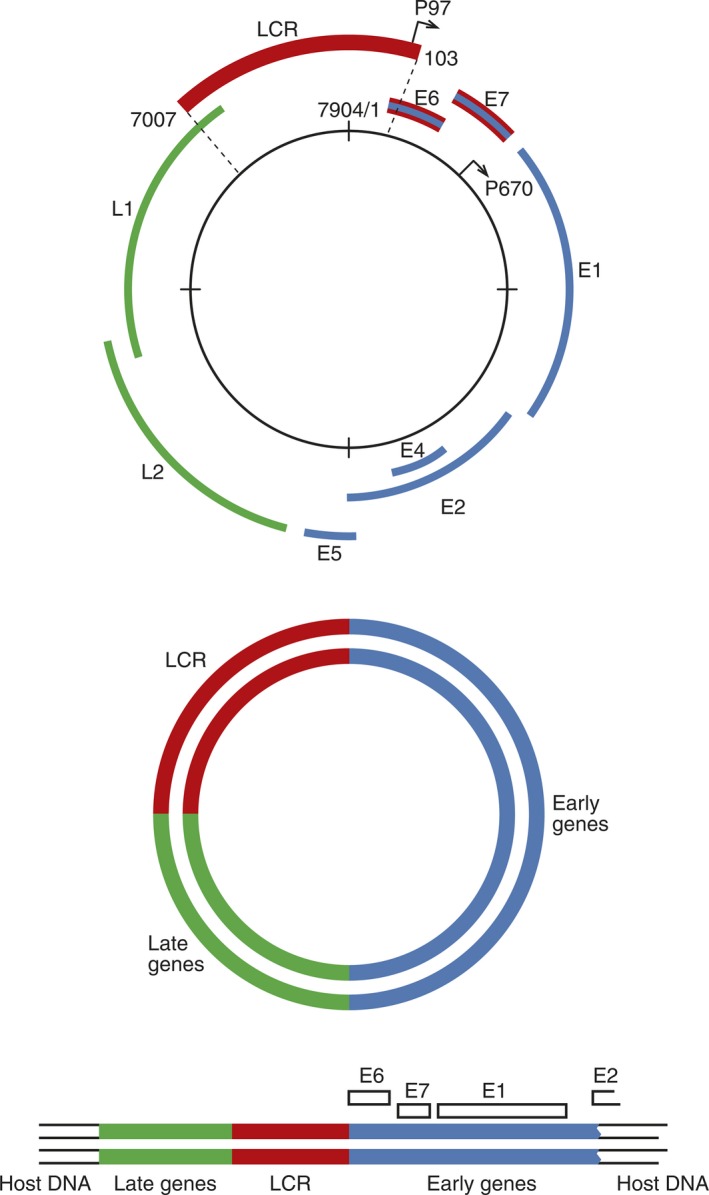
Organization of the human papillomavirus (HPV) genome. Early genes (E1, E2, E4, E5, E6, and E7) code for non‐structural proteins, while late genes (L1 and L2) code for structural proteins. The long control region (LCR) contains a DNA replication origin and several binding sites for viral and host proteins to regulate viral DNA replication. The two major promoters P97 and P670 are marked. HPV can integrate into the host cell chromosome in a random pattern. During this integration, the double‐stranded circular DNA opens, which will disturb the function of the E2 gene, as given in the lower graph. Reprinted from Rautava J and Syrjänen S. Biology of Human Papillomavirus Infections in Head and Neck Carcinogenesis. Head Neck Pathol. 2012; 6(Suppl 1): 3–1. With permission from Springer Nature via Copyright Clearance Center's RightsLink service.

Human papillomavirus contains a circular double‐stranded DNA of approximately 8,000 base pairs that is enclosed by a non‐enveloped icosahedral capsid of 55 nm in diameter 2. Only one strand of the DNA genome is transcribed. This sequence contains 9–10 open reading frames (ORFs) with potential protein coding regions. The early (E) region contains ORFs encoding viral regulatory proteins and the late (L) region encodes the two viral capsid proteins L1 and L2 (Fig. 2). The third region of the genome is the long control region, also called the upstream regulatory region or the non‐coding region 2, 9. The long control region contains the origin of DNA replication and binding sites for both viral and host cellular proteins for controlling viral transcription (Fig. 2). Table 1 summarizes the ORFs and the function of the viral genes.

**Table 1:** Summary of human papillomavirus (HPV) open reading frames/genes and function of the viral proteins

Open reading frame/genes	Protein function
E1	Viral replication; mediates episomal DNA replication
E2	Regulates viral transcription and viral replication; regulates viral copy numbers
E4	Interacts with cytoskeletal proteins; facilitates virion release
E5	Downregulates MHC Class I molecules; stimulates cell proliferation; prevents differentiation; activates EGFR
E6	Maintains viral genome; inactivates several host‐cell proteins (e.g. inhibits apoptosis by degradation of p53, induces malignant transformation together with E7)
E7	Maintains viral genome; reactivates cellular replication mechanisms by binding pRB; induces malignant transformation together with E6
E8	Function is not totally known. A fusion protein 16‐E8^∧^E2 limits the viral copy number but is not required for plasmid persistence and maintenance
L1	Major viral capsid protein
L2	Minor viral capsid protein

EGFR, epidermal growth factor receptor; MHC, major histocompatibility complex; pRB, retinoblastoma tumor suppressor protein.

## HPV genotypes

Classification of HPVs is based on the nucleotide sequence of the ORF coding for the capsid protein L1 as this sequence is the most conserved among all HPVs. Human papillomavirus types with less than 60% homology within the L1 part of the genome belong to different genera. Different viral species within a genus share homology of between 60% and 70%. The HPV genotype is considered as novel if the homology of the L1 ORF to any other HPV type is less than 90% (Fig. 1) 6, 7. The novel HPV types are labelled in numerical order after the whole genome has been cloned and deposited in the International HPV Reference Center 6, 7. Currently, HPV‐16, in species 9 (Fig. 1), is the most powerful oncogenic human virus known.

## HPV infection

**Figure 3:**
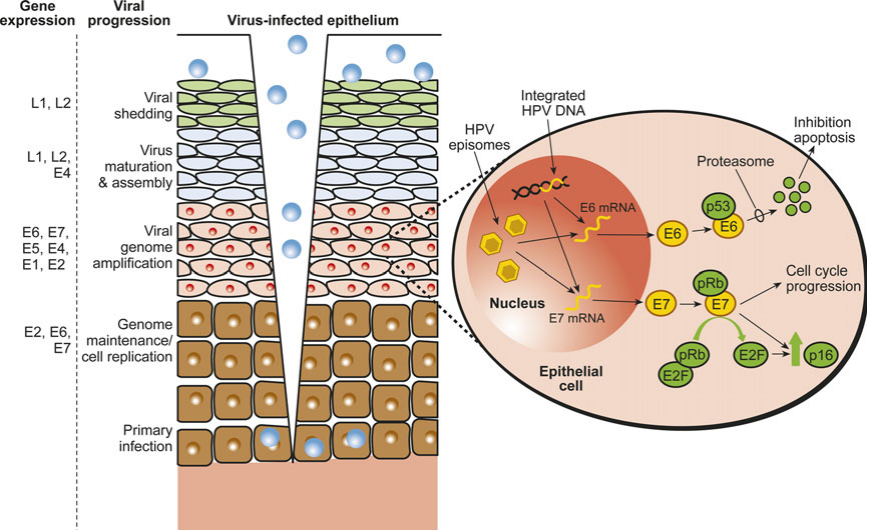
Human papillomavirus (HPV) infects the basal cells of the stratified squamous epithelia which become exposed after wounding or micro trauma. Virus particles are produced only in the surface of the epithelium, from where they are shed into the surroundings to infect new target cells. In the nuclei of basal cells, the viral genome remains as a low‐copy plasmid. Expression of viral proteins is regulated by differentiation of the infected cells during their movement toward the epithelial surface. At an early stage of the infection, viral DNA is replicated, along with cellular DNA, during cell division at S‐phase. At some point, a switch from stable replication (genome maintenance) to vegetative viral DNA replication will occur to allow the production of genomes for packaging into virions encapsulated by L1 and L2. Thousands of virus particles are produced per cell. After viral integration of high‐risk HPVs, the expression of E6 and E7 genes are permanently upregulated. HPV E6 binds and degrades p53, which leads to inhibition of apoptosis. HPV E7 protein binds and degrades the retinoblastoma tumor suppressor protein, pRB, disrupting its interaction with the transcription factor E2F. The release and activation of E2F results in expression of S‐phase genes and cell‐cycle progression. Upregulation of p16 is induced by HPV‐mediated disruption of E7, which leads to cellular accumulation of p16.

Human papillomaviruses have a tropism for the squamous epithelium 10. Viral particles infect the basal cells of the epithelium, which are exposed through micro‐abrasions or epithelial wounding (Fig. 3). The receptors of HPV and the mode of viral entry into the cell are still partly unknown. To this end, most data have been gathered from HPV‐16. In the extracellular matrix or basement membrane, HPV‐16 transiently binds to laminin 332 and/or heparan sulfate proteoglycans (HSPGs). Growth factor receptors on the cell surface may also become activated through the growth factor–HSPG–HPV‐16 complex. Endocytosis occurs via a macropinocytosis‐like mechanism involving actin. The virus trafficks through the endolysosomal system and the major capsid protein, L1, will mostly be dissociated from the viral genome–L2 complex, facilitated by cyclophilins 10, 11. The minor capsid protein, L2, mediates delivery of the viral genome to the epithelial cell nucleus. L2 associates with sorting nexin‐17, and interacts directly with sorting nexin‐27 to aid in viral trafficking. Entry of the viral genome into the nucleus requires mitosis and this process is mediated by L2. Following nuclear entry, L2 and the viral genome co‐localize at interchromosomal multiprotein aggregates, collectively called nuclear domain 10, which is a critical step in the establishment of an active infection and allows transcription of the viral genome 11.

The HPV life cycle is closely associated with the differentiation program of the infected host squamous epithelium in a still‐unknown way. Thus, HPV cannot be produced in vitro, which has hampered detailed dissection of the life cycle of this virus. As a consequence, there are still no virological definitions for a productive, chronic, or latent (so‐called persistent) HPV infection, making comparison of different clinical trials difficult. The schematic presentation of viral progression and gene expression in differentiating squamous epithelium is summarized in Fig. 3.

During the HPV life cycle, E1 and E2 are among the first viral proteins to be expressed. Viral DNA is maintained in basal epithelial cells as a stable multicopy plasmid or episome. E1 may not be necessary once the viral copy numbers have reached a threshold of 50–100 copies. Viral genomes replicate in cell division during the S‐phase, ensuring persistent infection of basal cells. In this ‘latent’ phase of the viral life cycle, HPV genomes are thought to persist in basal epithelial cells for years to decades. However, at some point, a switch from stable replication (genome maintenance) to vegetative viral DNA replication must occur to allow the production of genomes for packaging into virions. The mechanism regulating this switch is still unknown. E4 is embedded within the E2 gene and is primarily expressed as an E1^∧^E4 fusion protein during the late stages of the HPV life cycle. E4 binds to cytokeratin filaments, disrupting their structure, and is thought to play a role in viral escape from the desquamated epithelium (for review see ref. 2).

E5 is an oncogenic small, hydrophobic, single‐pass transmembrane protein that forms dimers and interacts with and activates receptor tyrosine kinase receptors, including the epidermal growth factor (EGF) and platelet‐derived growth factor (PDGF) receptors. Human papillomavirus E5 proteins play a role in apoptosis and also in evasion of the immune response. Human papillomavirus E6 and E7 proteins both drive cell‐cycle entry to allow genome amplification in the upper epithelial layers. High‐risk HPV E6 proteins have oncogenic activities. They bind and degrade p53, as well as cellular host signaling proteins with protein–protein interaction domains (i.e. PDZ proteins), and they activate telomerase (Fig. 3). The high‐risk HPV E7 proteins bind and degrade the retinoblastoma tumor suppressor, pRB, and contribute to the progression of malignancy by inducing genomic instability (Fig. 3) 2, 12, 13.

## HPV carcinogenesis

During carcinogenesis, the HPV genome frequently integrates into a host‐cell chromosome. Integration is random, and each site is unique depending on how and where the virus integrates 14. During the integration, expression of the transcriptional repressor, E2, is often lost. Persistent expression of E6 and E7 is necessary for maintenance of the transformed phenotype of carcinoma cells. The transforming activities of the high‐risk E6 and E7 oncoproteins is related to their ability to associate with, and dysregulate, several cellular regulatory protein complexes, of which the most important are p53 and pRB [Fig. 3; reviewed in 2, 12, 13]. E6 and E7 are capable of immortalizing keratinocytes but the immortalized cells are not tumorigenic. Thus, in HPV‐induced carcinogenesis, additional cofactors are needed. This has recently been studied by using HPV‐16 E6 and E7 transgenic mice in which the individual HPV‐16 oncogenes are expressed in the stratified squamous epithelium by using the human keratin 14 (K14) promoter 15, 16. Exposure to low levels of estrogen for 6 months resulted in the development of cervical carcinoma in these mice, but in oral mucosa, only aberrant epithelial proliferation was found. In the presence of the chemical carcinogen, 4‐nitroquinoline 1‐oxide (4NQO), HPV‐16 E6 and E7 transgenic mice developed oral and esophageal carcinoma, but also benign papillomas. Recently, it was shown that the cutaneous HPVs (*Betapapillomaviruses*) can also have an impact on oral carcinogenesis 17. After treatment with 4NQO, HPV‐49 E6/E7‐Tg mice were highly susceptible to upper digestive tract carcinogenesis, upon initiation with 4NQO, to the same extent as HPV‐16 E6/E7‐Tg mice, whereas no effect was seen in HPV‐38 E6/E7‐Tg mice 17. Thus, the animal studies mimic the progression of high‐risk HPV‐positive lesions toward malignancy, a process that occurs with a low frequency and requires the acquisition of additional host cellular mutations. Hence, HPV E6/E7 oncoproteins mechanistically contribute to the initiation and progression of cancer [reviewed in 12, 13].

## Transmission of oral HPV infection

Cutaneous warts caused by *Betapapillomaviruses* (HPV) are common in children, in whom they are known to be acquired mostly through horizontal transmission. Cutaneous HPV infections can persist asymptomatically for years in children, and even in newborns 18. Unlike the HPV infections in skin, mucosal HPV infections have been regarded as sexually transmitted infections, despite the fact that *Betapapillomaviruses* (HPV) have also been found in virgins, infants, and children, in both oral and genital mucosa. This implies a non‐sexual mode of transmission 19, 20, 21, 22, 23. Perinatal transmission has been regarded as the most likely explanation for the presence of HPV in newborns. Several studies have shown that children born to HPV‐positive mothers have a higher risk of becoming HPV positive 24, 25, 26, 27, 28, 29, 30, 31. A meta‐analysis on 3,128 mother–child pairs showed that children of HPV‐positive mothers were 33% more likely to be HPV positive than were children born to HPV‐negative mothers. This risk was even higher (45%) when only high‐risk HPV infections were considered 22. It was estimated that vertical HPV transmission was the most likely mode of virus acquisition in 20% of these children, and other plausible explanations included higher infection rate during early nursing from a mother to her child.

In our Kuopio cohort (1981–1998), we showed, several years ago, that vertically transmitted HPV was detectable for a period ranging from 2 d to 3 yr in 44% of the infants whose nasopharyngeal aspirate fluid tested HPV‐positive at birth. The overall concordance between HPV types found in the mother's genital sample and her infant's nasopharyngeal aspirate fluid was 69% 25, 26. Among 98 children, 0.3–11.6 yr of age, born to 66 mothers of this same cohort, HPV was detected in 32% of the oral scrapings, and 52% of these had the same HPV types as in the genital sample of the mother at the time of delivery, with HPV‐16/18 being the most prevalent type (81%) 25. This indicates that HPV can be acquired at early age and might remain latent for years.

In our Finnish Family HPV Study, we showed that HPV DNA was detected in 17.9% of baseline oral samples from newborns and in 16.4% of the maternal cervical samples. The at‐delivery HPV genotype‐specific concordance between the newborns and their mother was almost perfect (weighted kappa = 0.988). We also showed earlier that oral HPV carriage in newborns was highly significantly associated with HPV presence in the placenta and/or cord blood 30. Recently, trottier *et al*. 32 published their first results from the HERITAGE study on perinatal transmission and risk of HPV persistence in children. The design of that study is nearly identical to that of our cohort study. Their preliminary results on 167 women and their 67 infants sampled at birth or the 3‐month visit showed that the proportion of HPV positivity (any site) in children was 11%, with a range from 5% to 22%. They also showed that HPV was detectable in multiple sites (oral, pharyngeal, and genital) of the children, including the conjunctiva (5%) 32.

These data strongly support the hypothesis that HPV can be transmitted vertically and cause a true infection of the newborn's oral mucosa. In a proportion of these children, oral HPV infections acquired at birth can persist for years without development of any significant clinical lesions. Importantly, these early childhood infections also represent an active infection because HPV‐specific cell‐mediated immunity (CMI) is seen in these sexually inexperienced children 33. These observations support our hypothesis that oral mucosa is a common site for the first exposure to HPV and may explain why half of healthy adults demonstrate HPV‐specific CMI, irrespective of their partner/sexual status 35.

We have also reported that oral sex does not predict the partner's oral HPV status. Rather, horizontal HPV infection (from mouth to mouth via saliva) could be a mode of HPV transmission as persistent oral HPV infection of a person was predicted by the presence of persistent oral HPV in his/her spouse (OR = 10.0, 95% CI: 1.5–68.7, *P* < 0.005) 35. Our findings indicate that transmission may occur via saliva. However, d'souza *et al*. reported that oral sexual behavior was the primary predictor of oral infection with HPV‐16. However after adjusting for, age cohort and race, oral sex was no longer associated with oral HPV 36.

## Asymptomatic oral HPV infection

Human papillomavirus DNA is found in the mouth but the frequency of asymptomatic infections in which HPV DNA is detectable without any clinical lesion is still a matter of debate, as evidenced by kreimer *et al*. 37, sanders *et al*. 38, rautava *et al*. 39, and kero *et al*. 40. kreimer *et al*., in their review, reported that asymptomatic oral HPV‐16 infection and any HPV type was found in 1.3% and 4.5% of the nearly 4,000 subjects included in the review 37. sanders *et al*. 38 reported that in the USA, oral asymptomatic HPV infection is three times more prevalent in men (11%) than in women (3.7%). They also described a bimodal pattern of oral HPV infection in the age groups 25–30 yr and 55 yr (oral HPV prevalence at highest in 17% of the studied population), but this was detectable only in male subjects. A substantially higher risk of HPV infection in men was not explained by education, smoking, age of sexual debut, or number of lifetime sex partners 38. In our Finnish Family HPV Study cohort, oral HPV prevalence during the 6‐yr follow‐up varied from 15% to 24% in women and from 15% to 31% in their male spouses 39, 40.

In children, a wide variation (of 4–87%) is seen in estimates of oral HPV prevalence**.** Interestingly, a bimodal pattern, with peak prevalences at <1 yr of age and 13–20 yr of age, has been found. There is growing evidence that HPV infection can be transmitted early in life by vertical transmission from mother to child 20, 22, 41, 48, 49. This transmission could occur during fertilization, during pregnancy (prenatal) or delivery, or in the early months of the newborn's life (perinatal) 20, 21, 41. There is evidence that children of HPV‐positive mothers are at 33% higher risk of becoming HPV positive during the first weeks after birth than are children of HPV‐negative mothers 22.

## Wide variation in oral HPV detection: why?

The wide variation in detection rates of asymptomatic oral HPV can be explained by three main variables: (i) the sampling site; (ii) the sampling method; and (iii) the HPV testing methods. In most studies, the exact anatomic sites of the samples are not given and thus the origin of HPV infection (whether oral or oro‐pharyngeal) is impossible to trace. Samples are obtained as mucosal scrapings, oral rinses, or tissue biopsies. There are two types of normal oral mucosa: keratinized and non‐keratinized. When a scraping is taken from an orthokeratinized mucosa (similar to the skin), the sample is frequently inadequate because of a lack of nucleated cells. In addition to the HPV genotype coverage, sensitivity and specificity of the HPV testing method, as well as the transport medium, are of key importance. Based on our method validation study on HPV testing from 309 scrapings, we found that three sequential swabs from the buccal mucosa will result in approximately 100,000 cells, which is optimal for HPV testing 42, 43. In these samples, HPV detection rate was related to the analytical sensitivity of the HPV testing method; HPV DNA was found in 3.8%, 15.6%, and 23.1% of samples using dot‐blot hybridization, Southern blot hybridization, and PCR, respectively 42, 43. An important aspect frequently neglected is the quality of DNA for PCR, particularly because the microbe load in the saliva is very high. One milliliter of saliva might contain 10–100 million microbes. This is specifically relevant when gargle or saliva samples have been used for HPV testing and they have been kept at room temperature for hours before analyses. Consequently, the extracted DNA might be mostly bacterial DNA, masking the few viral copies of DNA present in the samples. Accordingly, we have always taken oral brush samples into 80% alcohol and stored them at −80°C to prevent bacterial growth. Purified DNA will result in a much higher HPV detection rate than non‐purified DNA, as shown by us 20 yr ago 25, 26 and confirmed by D’souza *et al*. in 2005 44. Nested PCR will increase the HPV detection rate and should be used especially when the cell count in the sample is not optimal or the viral load/viral copies per cell is expected to be very low. In our Finnish Family HPV Study, we used nested PCR with subsequent hybridization to identify high‐ or low‐risk HPVs as two separate groups or for HPV genotyping. With this method, we found high‐risk HPVs in 16–27% of the oral scrapings taken into 80% ethanol. DNA extraction was performed using the high‐salt method 27, 39, 40, 45, 46. Similarly, kay *et al*. 47 reported that the HPV detection rate in buccal swabs increased from 19% to 74% when single PCR with MY09/11 primers was replaced with nested PCR with MY09/11 and GP05+/06+ primers.

## The Finnish family HPV study

**Figure 4:**
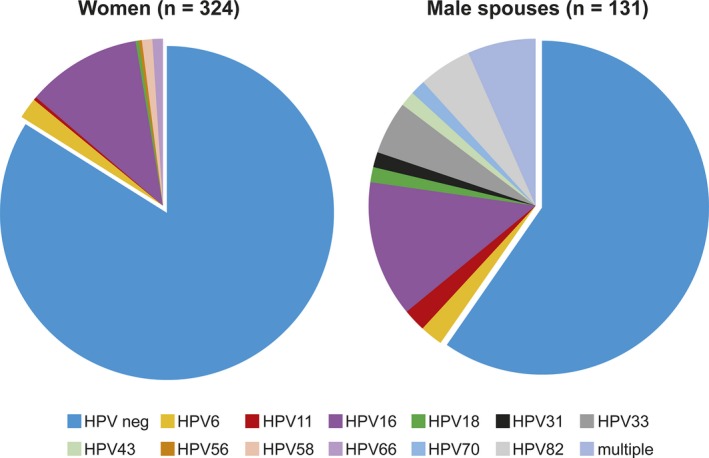
The prevalence of asymptomatic human papillomavirus (HPV) in oral brush samples taken from healthy young couples at entry to the Finnish Family HPV Study. In total, 17% of the oral samples in women tested HPV DNA positive, while 18.7% of the oral samples taken from their male spouses were HPV DNA positive. The HPV genotype distribution among the HPV‐positive samples is presented in the pie charts. The outcome of oral HPV infection in women and their male spouses during the 6‐yr follow‐up is given in references 39, 40, 51, 54. neg, negative.

The Finnish Family HPV Study was started in 1998, with the author as the principal investigator, to understand the dynamics of HPV infection within healthy young Finnish families. This study (still ongoing) is a continuation of our previous studies on HPV in oral mucosa and its transmission modes 25, 26, 42, 43, 48, 49, 50. We initially performed a follow‐up, for 36 months, of 331 pregnant women (mean age 25.5 ± 3.4 yr) at their baseline visit, their 131 spouses (mean age 28.8 ± 5.0 yr), and their 333 newborns 45. Subsequently, the follow‐up was extended to 6 yr, and over 50% of the cohort was compliant with that. Later a subgroup of mother–child pairs was recalled to study the cell‐mediated immunity of HPV‐16 in more detail. The study protocol included a detailed medical history and questionnaire on sexual habits, in addition to extensive serial sampling starting from the baseline visit before delivery, at delivery, and at 2, 6, 12, 24, 36, and 72 months postpartum, including samples of sera, saliva, placenta, breast milk, and semen. Our results (tested by nested PCR) showed that the prevalence of oral HPV varied from 15% to 24% in women and from 15% to 31% in their male spouses during the 6‐yr follow‐up with seven samplings at maximum 39, 40, 51. Nearly 20 different HPV genotypes were found, with HPV‐16 being the most prevalent. The genotype distribution and the prevalence of oral HPV in 229 women and their 131 spouses at baseline (when entering the study) are summarized in Fig. 4 39, 40, 51.

**Figure 5:**
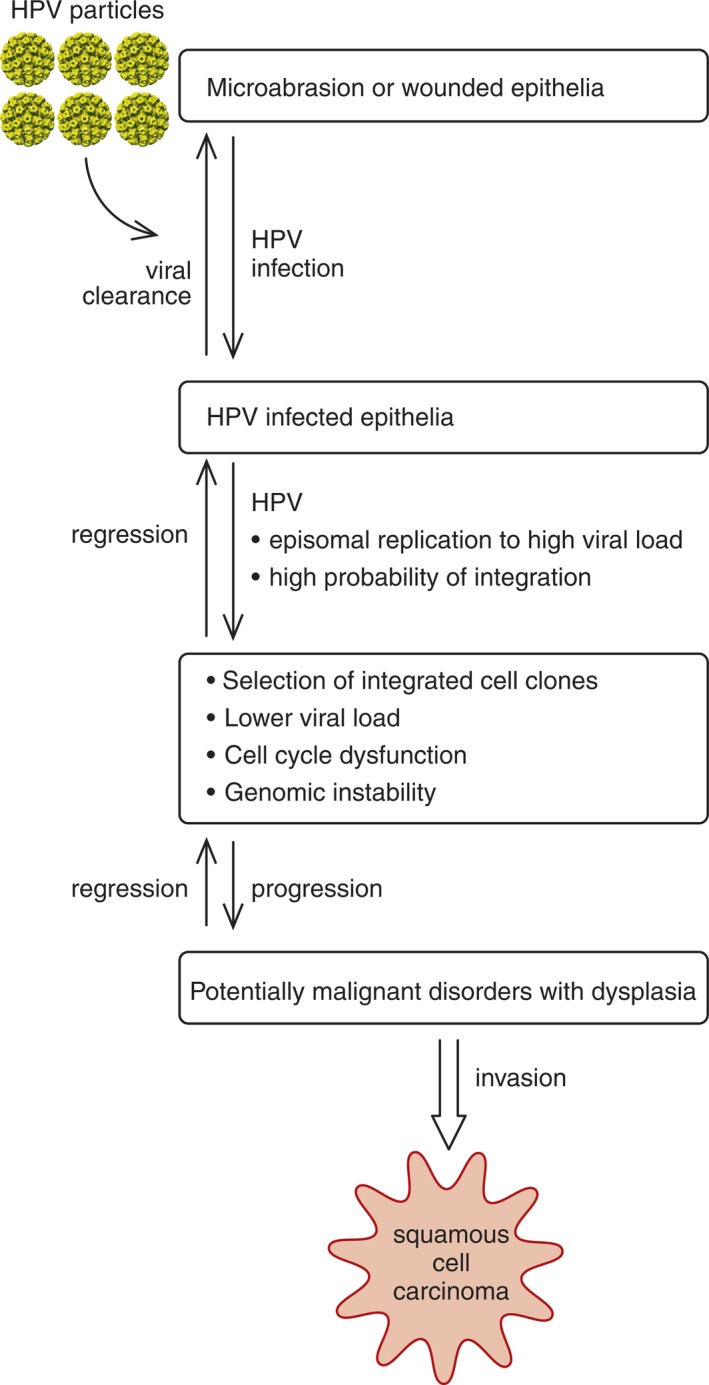
Schematic presentation of the stepwise progression of a human papillomavirus (HPV)‐infected cell toward carcinogenesis. Reprinted from Rautava J and Syrjänen S. Biology of Human Papillomavirus Infections in Head and Neck Carcinogenesis. Head Neck Pathol. 2012; 6(Suppl 1): 3–1. With permission from Springer Nature via Copyright Clearance Center's RightsLink^®^ service.

In our Finnish Family HPV Study, the prevalence of HPV DNA in the oral mucosa of infants fluctuated from 14% at delivery to 21% at 6 months, and reached a low of 12% at 36 months 27, 45. During the 6‐yr follow‐up, the prevalence of oral HPV in the offspring varied from 8.7% to 22.8% over time, and 18 HPV genotypes were identified. These values represent oral HPV infections and not oropharyngeal HPV, because all scrapings were taken only from the oral mucosa. During the 3‐yr follow‐up, 42% of the infants acquired incident HPV infection, while 11% cleared their HPV, and 10% had persistent oral HPV infection. No differences were found in gender distribution of oral HPV. Persistence of oral HPV in infants was associated with the oral HPV status of both parents, hand warts in mothers, young age of the mother at the onset of sexual activity, and the use of oral contraceptives by mothers 27, 45. Interestingly, our recent results showed that children who were born to mothers with incident cervical intraepithelial lesions (CIN) already had HPV‐16‐specific CMI 33. All of these children were sexually naive and were not vaccinated against HPV. Accordingly, HPV infections at an early age might affect the outcome of genital or oral HPV infections later in life.

## Persistent/chronic HPV infection is a risk for malignant transformation

The natural history of oral HPV infection is incompletely understood because only a few follow‐up studies are available and the follow‐up periods are still short 27, 36, 39, 40, 46, 51, 52, 53. Most oral HPV infections by the *Alphapapillomaviruses* do clear but a minority remain persistent. The HPV‐16 genotype is the most common HPV genotype to persist in oral and oropharyngeal mucosa 39, 40, 51, 52, 53. Our Finnish Family HPV Study showed that HPV infection can persist in 14% of healthy men 54. Smoking increases the risk for oral HPV persistence in men, while previous genital condyloma seems to be protective 40, 54. Oral HPV persistence (i.e. at least two consecutive samples positive for the same HPV genotype) was detected in 22% of the women 39. The use of oral contraceptives and new pregnancy protected from oral HPV, while smoking increased the risk for HPV persistence 39, 51. In line with our results, D’souza *et al*. reported that oral HPV infection clearance was reduced among male subjects [hazard ratio (HR) = 0.54; 95% CI: 0.37–0.78). This was affected by recent alcohol use (HR = 0.62; 95% CI: 0.42–0.91). Men were 37% less likely than women (adjusted HR = 0.63; 95% CI: 0.42–0.95) to clear their oral HPV during a (median) follow‐up of 12.2 months. No association was found with oral sex 36. Stable marital relationship protected men from oral and genital HPV infection 55.

Human papillomavirus 16 might become integrated into the host genome also in oral epithelial cells and this might happen even earlier during the infection than in the genital tract 12, 13, 14. Recently, we analyzed the physical state and viral copy numbers of HPV‐16 in asymptomatic oral infections that either persisted or cleared during a 6‐yr follow‐up period. In both genders, persistent oral HPV‐16 infections were associated with the mixed or integrated form of HPV‐16, while in the clearance groups, episomal HPV‐16 predominated. This indicates that HPV‐16 integration is a common event, even in asymptomatic oral infections, which might predispose the infected subjects to progressive disease 56. It was also found that in women, persistent (>2 yr) genital HPV infection was predictive for impaired clearance of oral HPV infection 57. Taken together, our results suggest that long‐term cervical persistence of high‐risk HPV infection might be an indicator of a compromised host response to co‐existent oral HPV infections.

## HPV and immunosuppression

Immunosuppression as a result of organ transplantation or HIV infection is known to increase the risk for HPV infection 58, 59, 60. This increased risk of HPV infection in patients with HIV has been associated with an impaired immune response to HPV, highly active antiretroviral therapy (HAART), aging of HIV‐infected patients, and direct interaction between these two viruses 58, 59, 60. A recent study showed that HIV‐positive individuals had a twofold increased risk for oral HPV infection compared with HIV‐negative individuals. The overall HPV positivity in oral mucosa was 36%, and was 5.7% for HPV‐16 only 61. The predictors of oral HPV in HIV‐positive individuals included lower CD4 T‐cell count and higher number of lifetime sexual partners, while in HIV‐negative subjects, higher number of recent oral sex partners 61. Also, HPV‐induced lesions are more common in HIV‐infected subjects, especially when the patients are at a later stage of the disease and/or are under HAART 59. The recent paper by camacho‐aguilar *et al*. 62 showed that nearly 76% of the patients in their study, most of whom had developed AIDS and/or were under HAART, had more than one oral HPV lesion, mostly with florid presentation. Also, multifocal epithelial hyperplasia lesions were prevalent (62%) and were caused by HPV‐13 (26%) and HPV‐32 (31%). As expected, HPV viral loads were higher among individuals with multiple oral lesions than in those with solitary lesions 62. Importantly, HPV‐32 seems to be, in general, much more prevalent in asymptomatic oral HPV infections and warty lesions among HIV‐infected subjects than among the general population 63. The reason for this over‐representation as a single HPV‐32 infection or as part of multiple‐type infections is not known. Generally, it has been considered that the prevalence of HPV‐associated cancers increases in HIV‐infected subjects. However, beachler *et al*. 64 showed, in their cohort of 82,375 HIV‐infected participants, that lower CD4 cell count before cancer diagnosis was significantly associated with increased HPV‐related and HPV‐unrelated head and neck squamous cell carcinoma (HNSCC) risk. Thus, immunosuppression as such may have a role in the development of both HPV‐related and HPV‐unrelated HNSCC.

Regarding HIV genes, there is evidence of an interaction between HPV and *tat*,* rev*, and *vpr*. HIV might play a role in HPV‐associated pathogenesis by exhorting oncogenic stimuli via *tat* and *rev* or vice versa 63, 65.

## The role of gingival pocket epithelium as an HPV reservoir

As discussed above, HPV is detected asymptomatically in oral mucosa but the origin or reservoir of oral HPV is still unknown. The possible reservoirs in the mouth include inflamed gingival pocket, ductal epithelium of the salivary glands, cryptal epithelium of the tonsils, border of oral cavity, and oropharynx (i.e. the border of ectoderm and endoderm, as seen in the transformation zone in the uterine cervix). A reservoir could also be a latent HPV infection in epithelial basal cells, in which a switch from a stable replication (genome maintenance) to vegetative viral DNA replication can start after a local irritation, as shown in an experimental model in 1943 66.

As the gingival pocket is the only site in the oral mucosa in which basal cells, the known targets of HPV at other mucosal sites, are normally exposed to the environment, we hypothesized that this could be the site of a latent HPV infection in oral mucosa. Because local inflammation is nearly always present in the gingival pockets, epithelial cell division is enhanced, aiding viral replication 67. To support that view, we analyzed gingival samples from 31 patients with periodontitis. Upon analysis with PCR, high‐risk HPV types were detected in 26% (eight of 31) of the samples. More importantly, by using *in‐situ* hybridization, we were able to localize the viral DNA in the coronal part of the junctional epithelium of the gingival pocket, supporting our hypothesis that inflamed gingival pocket epithelium could be one possible reservoir of HPV infection. While having important implications in understanding HPV transmission, this observation did not rule out the possibility that HPV may be involved in the initiation of periodontal disease 67. Accordingly, tezal *et al*. 68 proposed that while periodontal infections create a suitable niche for oral HPV infections, it is also possible that oral HPV infections will exacerbate periodontal diseases.

This proposition has been investigated by comparing the presence of HPV in oral rinses with the patient's periodontal status. The US National Health and Nutrition Examination Survey data from 2009–2010 and 2011–2012 were combined, which allowed a study of over 6,000 individuals (30–69 yr of age) with clinically assessed periodontal status and HPV data. The adjusted OR for the presence of HPV in oral rinse specimens of subjects with periodontal disease was 1.04 (95% CI: 0.63–1.73), after adjustment for sex, race/ethnicity, education, age, income‐to‐poverty ratio, smoking, alcohol use, and number of lifetime sexual partners 69. In Australia, a study was performed on 223 participants with known oral hygiene and periodontal disease status and HPV‐positive oral rinse samples. Of these participants, 10 (4.5%) were HPV‐16 DNA positive according to PCR and Sanger sequencing. Within the HPV‐16 DNA‐positive subjects, seven (70%) and three (30%) were associated with poor oral hygiene and periodontal disease, respectively 70. Thus, a trend toward a positive correlation between oral HPV‐16 infection and poor clinical oral health status was shown.

To conclude, HPV can be found in the coronal part of the junctional epithelium in periodontal patients, but it is too early to draw any conclusions on its role in the pathogenesis of periodontal disease. However, some of the bacterial species important in periodontal disease could be cofactors in HPV persistence, which could explain why the gum is one of the most common sites of HPV‐associated oral squamous cell carcinomas (OSCCs). Further evidence that chronic periodontitis may be a significant factor in the natural history of HPV infection in patients with base‐of‐tongue cancers was provided by tezal *et al*. 71. They reported that every millimeter of alveolar bone loss was associated with an increased risk, of approximately fourfold, of HPV‐positive tumor status after adjustment for age at diagnosis, sex, race/ethnicity, alcohol use, smoking status, and number of missing teeth. The number of missing teeth was not associated with HPV tumor status 68.

## Benign HPV lesions

### Oral papillomas/condylomas

There are no population‐based studies on the incidence or prevalence of oral squamous cell papillomas (SCPs) or oral condylomas (COs). The largest cohort study comprises 20,000 Swedish citizens, 0.1% of whom had oral warty lesions 72. Squamous cell papillomas are the most common benign tumors of the oral epithelium. However, in some textbooks, papillomas are grouped together with benign epithelial hyperplasia, which are reactive changes to injury rather than true neoplasia. Squamous cell papillomas are reported to occur most frequently in children and in adults in their fourth and fifth decades of life. There are also some rare syndromes which are known to be associated with multiple oral papillomas, such as focal dermal hypoplasia syndrome, acrodermatitis enteropatica, Cowden syndrome, nevus unius lateralis, Costello syndrome, and Down syndrome (OMIM database; http://www.ncbi.nlm.nih.gov/omim OMIM).

### Oral condylomas

Oral condylomas have been traditionally associated with oral sex, in contrast to oral SCPs. However, there are no diagnostics (clinically or in histological examination) that can reliably differentiate oral SCPs from OCs. In the literature published to 1998, the author found a total of 481 SCPs and 284 OCs, of which 50% and 75%, respectively, tested HPV positive 73. Human papillomaviruses 6 and 11 were the major HPV types in these lesions 5, 20, 73, 74, 75, 76, 77, 78. The role of HPVs in the development of benign epithelial hyperplasia is unknown. Recently, some evidence was presented indicating that the majority of white oral mucosal lesions – flat, exophytic, wart‐like, or papillary proliferations – could be considered as clinical manifestations of HPV infection 79.

In our cohort of children born to mothers with genital HPV infection, we correlated HPV DNA status (PCR and oral scrapings) with the clinical appearance of their oral mucosa 25. Clinically, minor hyperplastic growths were found in 22.4% of these 98 children who had a mean age of 4.0 (range: 0.6–11.6) yr. Eight (36.4%) of these children with clinical findings tested HPV positive. Interestingly, a 7‐yr‐old girl had an HPV‐16‐positive oral SCP, and HPV‐16 was also detected in the genital tract of her mother at delivery 25.

There are very few studies on the infectivity of HPV‐positive oral papillomas or their clinical course. However, an excellent in‐vitro study, published in 1943, describes the natural history of oral papillomas in cottontail rabbits 66. Most probably, the behavior of human oral papilloma mimics that described in this early experimental study, which makes it worthwhile to review the study briefly here. Close contacts are known to be important in viral transmission. Accordingly, naturally occurring oral papillomas were found in 16.5% (119/722) of the domestic rabbits living in university animal houses but in none of 300 wild rabbits 66. The papillomas in domestic rabbits were located under the surface of the tongue and occasionally in the gums and on the floor of the mouth. Experimental transmission of oral papillomas was successful in 81.5% (66/81) of the rabbits using crude suspensions of Bekerfeld filtrates of ground papillomas. The papillomas developed after 6–36 (average 14) days, and they increased in size for about 1 month. Some papillomas persisted for as long as 400 d 66. The virus was unaffected when heated to 55, 60°C, or 65°C for 30 min but was totally inactivated when heated to 75°C or 80°C. Rabbits with papillomas that had already regressed or were regressing were resistant to reinfection. In some rabbits, the virus remained latent after clearance of the papilloma but could be activated simply by injuring or irritating the area. Viruses could be washed out of the mouths of rabbits carrying the growths and also from the mouths of rabbits with normal mucosa but carrying a latent infection. Transmission of virus from a mother to her offspring was shown, and there were ‘papilloma families’, in which transmission of virus from a mother to her offspring occurred via saliva during licking 66.

## Focal epithelial hyperplasia or Heck's disease

Focal epithelial hyperplasia (FEH) is a benign familial disorder with autosomal‐recessive inheritance 80. There is no gender predisposition and the lesions are found both in children and in adults. In oral mucosa, FEH is visible as multiple, whitish, soft and nodular elevations, which can disappear and recur. Focal epithelial hyperplasia is rare, and no reliable prevalence data exist at the population level 81. However, based on early studies, FEH is most prevalent among Native American and Mexican Indians and Indigenous peoples of South America and Eskimos and can be found in 35% of inhabitants in certain areas of Greenland 81, 82, 83. Focal epithelial hyperplasia has been associated with leukocyte adhesion deficiency 84, the human leukocyte antigen (HLA)‐DRB1*0404 allele 85, and recently also with mutations in adjacent transmembrane channel‐like protein (TMC) *TMC6* or *TMC8* (also called *EVER1* and *EVER2*) genes located on chromosome 17q25 86. These mutations are associated with epidermodysplasia verruciformis (EV), characterized by eruptions of wart‐like lesions that may occur anywhere on the body and are caused by skin HPV (*Betapapillomavirus*). On sun‐exposed areas, EV lesions will progress to squamous cell carcinomas 87. Transmembrane channel‐like proteins are transmembrane channel proteins which form a complex with the zinc transporter, ZnT1, thus regulating intracellular zinc distribution. In HPV‐infected cells, E5 protein of several mucosal genotypes (including HPV‐16) can bind to this complex, which increases the activity of transcription factors Ap1 and Zn+, allowing HPV replication 88.

## Treatment of benign oral HPV lesions

Among the treatments available for papillomas/condylomas, verrucas, and FEH are cryotherapy, electrosurgery, surgical removal, laser therapy, and trichloroacetic acid. lordyua *et al*. 89 showed that three applications (each of 30–60 s) of trichloroacetic acid resulted in atraumatic resolution of such lesions in a time span of 45 d. Imiquimod has been widely used to treat genital HPV lesions. mendoz‐flores *et al*. described a successful treatment of FEH lesions with topical 5% imiquimod cream applied every night for 2 wk. Mild erosion and ulceration developed in the upper labial mucosa, which were managed with lubrication (petrolatum ointment). After 2 wk, all of the small lesions had disappeared and the largest plaque resolved 1 wk later 90.

## Oral squamous cell carcinoma

Oral carcinogenesis is a multifactorial process involving socio‐economic, environmental, and microbial risk factors, leading to multistep changes. Smoking and tobacco explain nearly 80% of OSCCs 91, 92. Importantly, smoking and tobacco exposure seem to modify the survival and recurrence of HPV‐positive HNSCCs, especially oropharyngeal squamous cell carcinoma (OPSCC), and this should be considered in all future trials for risk stratification of HPV‐positive patients, as discussed later in this text.

In 1975, ZUR HAUSEN suggested that HPV is involved in cervical carcinogenesis 93. Seven years later, in 1983, we published the first evidence suggesting that a subgroup (some 20%) of OSCCs is associated with HPV, based on detection of HPV structural proteins in these lesions using immunohistochemistry with an antibody prepared against pooled HPV types 94. We subsequently identified HPV types 11, 16, and 18 in these samples 78, 95, 96. Since then, a huge number of studies have been published, repeatedly reviewed, and subjected to several meta‐analyses 96, 97, 98, 99, 100. One of the most cited works is a systematic review, by kreimer *et al*. 97, on the global distribution of HPV genotypes in HNSCCs. They identified 5,046 HNSCC specimens from 60 studies, with an overall HPV prevalence of 25.9%. The HPV prevalence was significantly higher in OPSCCs (35.6%) than in OSCCs (23.5%). ndiaye *et al*. 98 reported, in their meta‐analysis, that the prevalence of HPV in HNSCCs is geographically highly variable. In total, 148 studies were included, contributing data from 12,163 patients with HNSCC from 44 countries 98. Human papillomavirus DNA was detected in 3,837 cases. The pooled HPV DNA prevalence was 24.2% for oral cancer; the highest values were found in Asia (43.3%), in contrast to Europe, where the prevalence was 17.5%. Human papillomavirus 16 accounted for 82.2% (95% CI: 77.7–86.4%) of all HPV DNA‐positive cases 98.

In 2016, castellsagué *et al*. 99 reported their data on 3,680 formalin‐fixed, paraffin‐embedded cancer samples from the oral cavity, pharynx, and larynx derived from pathology archives in 29 countries. Human papillomavirus DNA was detectable in 24.9% of the oropharyngeal carcinoma samples. This value decreased to 18.5% when the criteria for HPV positivity was based on HPV DNA detection together with p16 positivity and expression of 16 E6*I mRNA. Human papillomavirus DNA positivity in oral carcinomas was found for only 7.4% of the samples, and only 3% of the samples were simultanously HPV DNA^+^, p16^+^, and 16 E6*I mRNA positive 99. One explanation for these lower HPV prevalence figures is the use of formalin‐fixed samples and the relatively small number of samples from each individual country.

In 2007, the 7th World Workshop on Oral Medicine launched a task force to find evidence for association of HPV with oral cavity carcinoma based only on case–control studies. A systematic literature search from January first 1966 to September 30th 2010, without language restriction, was performed 100. Initially, a total of 1,121 studies on HPV in OSCC and oral potentially malignant disorders (OPMD) were identified. Full texts were acquired for 62 studies. Of these, 39 met the criteria of a case–control design. The relative risks of HPV association with OSCC (1,885 cases and 2,248 controls) and OPMD (956 cases and 675 controls) were calculated separately. There was significant heterogeneity between studies (*I* ^2^ = 71%). The pooled HPV prevalence was 33.7% in the OSCCs and 12.0% in controls. The pooled OR across all studies was 3.98 (95% CI: 2.62–6.02) for an association between OSCC and HPV. Human papillomavirus‐16 was the most prevalent genotype detected. The pooled OR for association between OPMD and HPV was 4.1 (95% CI: 2.87–5.93). Among the subgroups, the estimates for HPV association were as follows: unspecified OMPD (OR = 4.3; 95% CI: 2.4–7.9); lichen planus (OR = 5.1; 95% CI: 2.4–10.9); leukoplakia (OR = 4.0; 95% CI: 2.3–6.9); and oral dysplasia (OR = 5.8; 95% CI: 1.5–22.3). Human papillomavirus‐16 associated with oral lichen planus and leukoplakia [OR = 5.6 (95% CI: 2.4–13.0) and OR = 4.5 (95% CI: 2.2–9.0), respectively] 100. This clearly underlines the importance of identifying the presence of HPV‐16 in these lesions because they are the most likely to progress.

Albeit the main focus of this paragraph is the association of HPV with oral cavity carcinoma, some key aspects of HPV in oropharyngeal carcinoma are also summarized. Human papillomavirus‐associated oropharyngeal carcinoma differs from other HNSCCs with respect to its epidemiology and disease outcome. The prevalence of HPV‐associated oropharyngeal cancer, especially tonsillar and base‐of‐tongue cancer, has increased significantly in Western part of the Europe and North America since the early 1970s 101, 102. Based on a meta‐analysis by dayyani *et al*. it has been estimated that, before 2000, 40.5% of oropharyngeal cancers were HPV associated, and that during 2000–2004 and 2005–2009, HPV was present in 64.3% and 72.2% of oropharyngeal carcinomas, respectively 103. Human papillomavirus prevalence in non‐oropharyngeal carcinomas (21.8%) has not increased over time. Recently, schache *et al*. reported that in the UK, the proportion of oropharyngeal carcinomas testing HPV positive remained unchanged during 2002–2011 104. These data were based on 1,602 patients collated from 11 centers 102. Similarly to findings in other studies, the HPV‐positive patients were found to be younger (69.2% below 50 yr of age) and more frequently male (54.3%). The most common site of the HPV‐positive oropharyngeal carcinomas was tonsils (61.8%), followed by the base of the tongue (49.4%).

The survival of patients with HPV‐positive oropharyngeal carcinomas is better than seen for any other HNSCC. The outcome of oropharyngeal cancer is related to HPV status, nodal metastasis, and other comorbidities. In addition, smoking and tobacco exposure seem to modify the survival and recurrence of HPV‐positive carcinomas 105. Accordingly, patients with oropharyngeal carcinoma can be classified into three survival categories based on their HPV and smoking status. The highest 5‐yr overall survival (OS) was found in patients who had HPV‐positive carcinoma and were never smokers (81%), followed by HPV‐positive patients who had ever smoked (48%); the lowest 5‐yr OS (21.1%) was found in HPV‐negative patients who were smokers. Recent studies support the view that HPV and smoking act as independent risk factors for oropharyngeal cancer. It has also been shown that the prevalence of oropharyngeal cancer increases with smoking for both HPV‐16‐positive and HPV‐16‐negative persons 106.

HPV‐associated tumors are also different from the smoking‐associated HNSCCs at genome level 107. Based on the results of the NIH Atlas Genomic Studies, HPV‐associated tumors are dominated by helical domain mutations of the oncogene *PIK3CA*, novel alterations involving loss of *TRAF3*, and amplification of the cell cycle gene *E2F1,* in contrast to the classical smoking‐related HNSCCs that demonstrate near‐universal loss‐of‐function *TP53* mutations and *CDKN2A* inactivation with frequent copy number alterations, including amplification of 3q26/28 and 11q13/22 105, 107. There is a subgroup of oral cavity cancers with favorable clinical outcome that display infrequent copy number alterations in conjunction with activating mutations of *HRAS* or *PIK3CA*, coupled with inactivating mutations of *CASP8*,* NOTCH1*, and *TP53* 107. One might speculate that these carcinomas could represent tumors in which HPV has been an initiator. Accordingly, HPV might integrate in early lesions, which results in permanent overexpression of HPV‐16 E6 and E7, aiding in immune evasion, inhibiting apoptosis, suppressing the expression of tumor‐suppressor and caretaker genes, and upregulating other oncogenes, such as *MYC* and *Ras*. We have characterized the stepwise early events in the progression of HPV‐induced lesions toward malignancy in the UT‐DEC‐1 cell line established from vaginal intra‐epithelial neoplasia with mild dysplasia (VAIN 1). Vaginal carcinoma mimics oral carcinoma in many respects, including the fact that only a subgroup is HPV associated. In the UT‐DEC‐1 cell line during the passaging from 7 up to 185, we could identify events previously shown to be important in the progression to malignancy: (i) virus integration into the cell genome and episome loss; (ii) selection of cells with an acquired growth advantage and ability to maintain telomerase activity; and (iii) a final stage of malignancy with permanently upregulated telomerase 108, 109, 110, 111.

## HPV and immunity

During HPV infection, the replicating virus does not kill the host cell and therefore viral antigens are not presented and inflammation is not induced. Human papillomavirus escapes immune attack through a number of mechanisms, which include (i) establishment of immunological ignorance, (ii) modulation of apoptosis, (iii) dysregulation of interferon responses, (iv) perturbation of antigen processing and presentation, (v) modulation and trafficking of antigen‐presenting cells, and (vi) polarization of T‐cell phenotypes. The microenvironment of keratinocytes is modified mostly by E6 and E7 proteins but also by E5 using several strategies 112, 113, 114, 115, 116, 117. E7 protein inhibits the expression of major histocompatibility complex (MHC) Class I proteins on the cell surface. E5 protein also downregulates MHC/HLA Class I, but acts selectively on the surface expression of HLA‐A and HLA‐B, which present viral peptides to MHC Class I‐restricted cytotoxic T lymphocytes, but not the natural‐killer cell inhibitory ligands HLA‐C and HLA‐E 117. Moreover, HPV can avoid immune recognition by downregulating toll‐like receptor 9 by E6 and E7 proteins, which results in non‐response to danger signals 118. E6 and E7 proteins also downregulate interferon expression and interferon‐dependent pathways, thereby preventing the activation of T cells 119. E6 upregulates the expression of interleukin‐10 and interleukin‐17. E6 downregulates expression of E‐cadherin, which leads to weak adhesion between keratinocytes and Langerhans cells, blocking their migration to the lymph node 120.

E5 plays an important role in escaping both innate and cell‐mediated immunity 117, 121. The bridging of innate and adaptive immune responses can be affected by E5 via the inhibition of CD1d‐mediated cytokine production 121. Thus, the lack of costimulatory signals by inflammatory cytokines, including interferons, during antigen recognition may induce immune tolerance rather than the appropriate responses. During a chronic HPV infection there will be a shift from a balanced T‐helper cell 1/T‐helper cell 2 ratio toward a T‐helper cell 2 and/or T2 cell + T‐helper cell 17 phenotype. Similarly, regulatory T‐cells are induced. In the future, potential new therapies of HPV infection could target the prevention of HPV entry into the cell, as well as interventions against HPV immune escape 122, 123, 124, 125.

As discussed before, the results of our Finnish Family HPV study show that HPV infections are present in sexually naïve children. This raised the question about the role of early HPV infections in either protecting from or predisposing to further HPV infections. We therefore studied HPV‐16‐specific CMI in 10 children born to mothers with an incident CIN diagnosed during a 14‐yr period and in 21 control children born to mothers who remained HPV negative 125. All children, except two controls, displayed CMI against HPV‐16 E2, E6, and/or E7 peptides associated with type 1 and 2 cytokine secretions. In total, 50% and 57% of the cases and controls, respectively, had HPV‐positive oral samples at at one or more follow‐up visits over a 6‐yr period. The children who did not have any maternal HPV antibodies during the first 6 months post‐partum showed more frequent proliferative responses of peripheral blood mononuclear cells after exposure to HPV‐16 than did the other children (*P *=* *0.045) 33, 124, 125. To conclude, HPV‐16‐specific CMI is common in young, sexually inexperienced children, and oral HPV infections are frequently seen in young children. The T‐helper 2 cell cytokine profile might also predispose to persistent oral HPV‐16 infection in women 34, 125 ***.*** We recently showed that the HLA‐G allele concordance might affect the vertical transmission of HPV, especially to the oral cavity 126, 127. We showed that the HLA‐G genotype concordance of G^∗^01:01:01/01:04:01 increased the risk of positivity for the high‐risk HPV genotype in umbilical cord blood and infant's oral mucosa. The mother‐to‐child concordance of G^∗^01:01:02/01:01:02 increased the risk of positivity for oral HPV with high‐risk HPV genotypes, both in the mother and the offspring, with an OR of 2.45 (95% CI: 1.24–4.85).

## HPV diagnosis

Generally, there are several methods available for routine viral diagnosis. These include viral cultures, detection of pathognomonic (cytopathic) cellular changes caused by the virus, and detection of viral proteins or nucleic acids in the samples or antibodies in the sera. Unfortunately, HPV cannot be cultured. For many years, routine light microscopy was almost the only method used to identify HPV in the biopsies. In light microscopy, the cytopathic cellular changes caused by HPV include koilocytosis, multinucleation, dyskeratosis, and parakeratosis, and these can be reliably detected in cytological smears [e.g. in PAP (cervical) smear] or in biopsies taken from the uterine cervix 128. Unfortunately, vacuolated epithelial cells are frequently seen in oral samples (associated with mechanical irritation) and this might obscure the identification of true koilocytes 50. The presence of HPV can also be identified by detecting viral antigens or nucleic acids in routine microcopy but the wide variety of HPV genotypes precludes rapid HPV testing. Previously, routine immunohistochemistry was based on identification of HPV L1 protein because it is the most conserved among all HPV genotypes. However, detection of L1 protein is reliable only in productive (vegetative) infections. p16 has been regarded as a surrogate marker for HPV infection. The concordance between p16 expression (detected by immunohistochemistry) and HPV DNA seems to be strongest in tonsillar cancer followed by tongue cancer. However, p16 expression is not reliable for estimating HPV positivity in oral samples, in other HNSCC samples, or in any of the benign or dysplastic oral lesions. It has recently been shown that using p16 positivity as a sign of HPV would result in approximately 20% of the HNSCC cases being false positive for HPV 105, 107, 129, 130.

Currently, HPV infection can be identified reliably only by detecting the viral DNA or RNA in the samples 131. Human papillomavirus positivity indicates the presence of the virus in the sampled area but nothing on the status of HPV infection or on past HPV infections or their outcome. Cytology combined with HPV DNA testing has been discussed as a sensitive method to detect patients at risk for HPV precancers and malignancies in the head and neck region but there is, so far, no consensus 132, 133. Over 200 commercial kits are available to detect HPV DNA or RNA 134. *In‐situ* hybridization methods are specific because the HPV‐positive signals can be localized and tissue morphology is simultaneously visualized. The current HPV diagnostics is mostly based on PCR‐based methods 131.

At present, there are no indications for screening for asymptomatic oral HPV infections because the natural history is incompletely understood. However, if the biopsied sample shows any HPV‐suggestive morphological changes, the presence of high‐risk HPVs, and specifically HPV‐16, should be excluded by in‐situ hybridization, using any of the commercial kits. The newest test – RNAScope – is a sensitive and specific test for detecting mRNA of 16 high‐risk HPV types 135. If these methods are not available, immunohistochemistry could be used for the simultaneous detection of the markers p53 (should be negative), D1 (highly expressed), p16 (positive), and Rb (negative) to determine the HPV infection.

Human papillomavirus serology has been disappointing in the diagnosis of genital HPV. Studies indicate that about half of patients with HPV‐positive cervical cancer have no HPV antibodies 136, 137, 138. There are only few studies in which the concordance between oral HPV infection and HPV serology has been assessed. In our Finnish Family HPV cohort, we found an association between oral HPV and for L1 protein among men but not among women 139, 140. We speculated that this is because in the male genitalia, the mucosa is scant and thus oral mucosa could be the site for immune recognition.

Several recent studies suggest that the presence of HPV‐16 E6 and E7 antibodies in sera can predict oropharyngeal cancer, even 5–10 yr in advance 141. Human papillomavirus 16 seropositivity conferred a greater than 14‐fold increased risk for subsequent development of oropharyngeal cancer. In a more recent study, 42% of the subjects diagnosed with oropharyngeal cancer between 1994 and 2009 in a US cohort were HPV‐16 E6‐seropositive, with stable antibody levels during the annual follow‐up examinations, which began 13 yr before diagnosis 142. Analysis of the primary tumors indicated that the sensitivity and specificity of HPV‐16 E6 antibodies were exceptionally high in predicting HPV‐driven oropharyngeal cancer. The HPV antibody status might also predict the disease outcome. E1‐, E2‐, and E6 antibody‐positivity was strongly associated with improved overall and progression‐free survival in the entire cohort as well as in patients with HPV‐16‐positive tumors 143. In the future, point‐of‐care HPV tests are likely to be the method of choice for identifying patients at risk for oropharyngeal cancer.

Human papillomavirus antibodies are also detected in saliva but their value in oral HPV diagnosis is not known. According to our unpublished data from the Finnish Family HPV cohort, both IgG and IgA to HPV‐16 L1 can be found in saliva of non‐vaccinated subjects. There was a correlation between salivary and serum antibody levels but only for HPV‐16 IgG. The patterns of HPV‐16 L1 IgG and IgA levels in saliva and sera were similar in women with persistent oral HPV infection and in those in whom oral HPV infection was cleared. Recent studies showed no statistically significant correlations between IgG in sera and saliva against HPV‐6 and HPV‐18. In contrast, the levels of HPV‐16 IgG in sera and in oral fluids were statistically significantly correlated 144, 145. hanna *et al*. 145 reported that HPV‐16 to early proteins was present not only in sera but also in saliva of patients with HPV‐associated OPSCC. In particular, E7‐directed antibodies were detected in saliva in the majority of patients and were associated with the HPV status. When analyzed longitudinally, the median levels of salivary E7 antibody decreased significantly post‐treatment. The authors’ conclusion was that measuring the levels of salivary E7 antibody at various time points may have utility in understanding HPV clearance and should be explored for their ability to predict the risk of recurrence 145. We have reasoned that the oropharynx could be the site of HPV immune recognition at any age, and that HPV‐16 E6/E7 antibodies could indicate a persistent HPV infection in that region with potential to progress toward malignancy, depending on the host factors and additional cofactors, as discussed previously in this text.

## Prevention of HPV infection

The discovery that the major capsid antigen L1 could self‐assemble into empty virus‐like particles (VLPs) was the starting point for the development of prophylactic VLP‐based HPV vaccines. It was soon demonstrated that L1 VPLs were highly immunogenic and protective against CIN lesions. At present, three commercially available prophylactic HPV vaccines exist: Cervarix, a bivalent (HPV‐16/18) vaccine (GSK, Middlesex, UK); Gardasil, a 4‐valent (HPV‐6/11/16/18) vaccine (Merck, Kenilworth, NJ, USA); and the recently launched Gardasil 9, a 9‐valent (HPV HPV6/11/16/18/31/33/45/52/59) vaccine (Merck). The licensed vaccines are safe and highly effective against the most common types of HPV found in cervical and other anogenital cancers. The extensive clinical trials with the bivalent (HPV‐16/18) and the 4‐valent (HPV‐6/11/16/18) vaccines have been successful. The implementation of vaccination programs in adolescent female subjects has been completed or is underway in many countries, but the impact of such programs critically depends on the population coverage and is improved by herd immunity. At the time of writing, HPV vaccination programs in adolescent men had been implemented in a few countries. roden & stern 146 have recently published an excellent review on the opportunities and challenges of HPV vaccination in cancer prevention.

The current key question is whether the HPV vaccines also prevent HPV infections in the head and neck region. By now it is known that HPV vaccination induces HPV‐specific antibodies in saliva 144, 146, 147, including neutralizing antibodies 144. The concentrations of HPV‐16 and HPV‐18 antibody were approximately 3 logs lower in saliva than in serum after HPV vaccination 147.

Therapeutic vaccines in humans have so far not been successful, even if experimental studies have been successful. However, vaccination with 13 synthetic long peptides (25–35 amino acids long) derived from the E6 and E7 oncogenic proteins of HPV‐16, together with nivolumab (Opdivo; Bristol‐Myers Squibb, New York, NY, USA), has been promising in treating HPV‐16‐positive oropharyngeal cancers 148. Nivolumab is a PD‐1 monoclonal antibody, and PD‐1 and PD‐L1 inhibitors block the immune‐suppressive effects of PD‐1, thereby allowing the immune system to attack and kill the tumor cells.

To conclude, as HPV antibodies can be detected in saliva, they will most probably be effective in preventing HPV‐induced head and neck diseases. The prophylactic vaccines should be taken before any HPV infection with the genotype present in the vaccine. Thus, the key question is the timing of vaccination, because some oral HPV infections are acquired during early childhood.

## Conclusions

Papillomaviruses are among the oldest viruses, being ubiquitous in animals and humans. During their long evolution, the most oncogenic HPV, HPV‐16, has acquired the capacity to hijack human cellular and immune systems to replicate and remain silent. The genotype distribution in asymptomatic oral HPV infection is similar to that found in the genital tract. The mother has a central role in infecting her offspring. The oral cavity might even be the first site of HPV entry into the human body. Oral papillomas/condylomas can be detected at any age, and as in the genital tract, they are caused by the most prevalent low‐risk HPV types, namely HPV‐6 and HPV‐11. Human papillomaviruses 13 and 32 do cause a specific clinical entity in oral mucosa – FEH or Heck's disease – which is not seen in the genital tract. A subgroup of HNSCCs is caused by HPV, mostly by HPV‐16. Oropharyngeal (tonsillar and base‐of‐tongue) cancers differ from the other HNSCCs in that (i) they are mostly caused by HPV, (ii) they have a better prognosis, and (iii) high levels of HPV‐16 E6/E7 antibodies can predict the development of cancer nearly 10 yr before it becomes apparent. Human papillomavirus antibodies can be detected in saliva, and their concentrations are increased after HPV vaccination. This supports the view that HPV vaccines can be protective also in the head and neck region, if given before the first exposure to HPV.
